# Serum levels of CTRP3 in diabetic nephropathy and its relationship with insulin resistance and kidney function

**DOI:** 10.1371/journal.pone.0215617

**Published:** 2019-04-22

**Authors:** Nariman Moradi, Reza Fadaei, Mohammad Ebrahim Khamseh, Ali Nobakht, Mohammad Jafar Rezaei, Fereshteh Aliakbary, Akram Vatannejad, Jalil Hosseini

**Affiliations:** 1 Department of Clinical Biochemistry, Faculty of Medicine, Kurdistan University of Medical Sciences, Sanandaj, Iran; 2 Endocrine Research Center, Institute of Endocrinology and Metabolism, Iran University of Medical Sciences, Tehran, Iran; 3 Sleep Disorders Research Center, Kermanshah University of Medical Sciences, Kermanshah, Iran; 4 Department of Nephrology, School of Medicine, Shahid Beheshti University of Medical Sciences, Tehran, Iran; 5 Department of Anatomy and Histology, Faculty of Medicine, Kurdistan University of Medical Sciences, Sanandaj, Iran; 6 Infertility and Reproductive Health Research Center, Shahid Beheshti University of Medical Sciences, Tehran, Iran; 7 Department of Comparative Bioscience, Faculty of Veterinary Medicine, University of Tehran, Tehran, Iran; East Tennessee State University, UNITED STATES

## Abstract

**Background:**

C1q TNF related protein 3 (CTRP3) is an adipokine secreted from adipose tissue. Previous studies have suggested that CTRP3 improves insulin sensitivity and reduces inflammation. Human studies have evaluated circulating levels of this adipokine in patients with diabetes mellitus (DM), diabetic retinopathy, metabolic syndrome, and coronary artery diseases. However, circulating levels of this adipokine in patients with diabetic nephropathy have not been evaluated. The present study aimed to assess serum levels of CTRP3 in patients with type 2 diabetes mellitus (T2DM) and diabetic nephropathy (T2DM-NP) and its relationship with metabolic and inflammatory markers.

**Methods:**

This cross-sectional study was performed on 55 controls, 54 patients with T2DM, and 55 patients with T2DM-NP. Serum levels of CTRP3, adiponectin, TNF-α, and IL-6 were measured by ELISA technique.

**Results:**

Serum levels of CTRP3 were significantly lower in patients with T2DM (257.61 ± 69.79 ng/mL, *p* < 0.001) and T2DM-NP (222.03 ± 51.99 ng/mL, *p* < 0.001) compared to controls (328.17 ± 80.73 ng/mL), and those with T2DM-NP compared to T2DM group. CTRP3 was independently associated with HOMA-IR (r = -0.327, *p* < 0.05) and adiponectin (r = 0.436, *p* < 0.01) in T2DM group. In T2DM-NP patients, CTRP3 independently was associated with eGFR (r = 0.428, *p* < 0.01) and HOMA-IR (r = -0.436, *p* < 0.01). Furthermore, CTRP3 revealed a ability to differentiate T2DM-NP patients from controls (area under curve (95% confidence interval): 0.881 (0.820–0.943) and *p* < 0.001).

**Conclusion:**

Decreased serum levels of CTRP3 in patients with T2DM and diabetic nephropathy and its association with pathologic mechanism in these patients suggested a possible role for CTRP3 in pathogenesis of diabetic nephropathy; nevertheless, further studies are required in this regard.

## Introduction

Adipose tissue is an active endocrine organ which secrets bioactive molecules called adipokine [1]. These molecules constitute a link between adipose tissue function and physiological and pathophysiological processes in the body such as glucose and lipid metabolism, endothelial functions, and inflammation [1]. Adipokines have shown to be potential biomarkers and therapeutic targets for diabetes mellitus (DM) and its complications [2].

Studies have revealed perturbation in the circulating levels of several adipokines in DM [3]. For instance, circulating levels of adiponectin diminish in patients with type 2 diabetes mellitus (T2DM) [4]. Adiponectin is the most abundant adipokine in circulation with favorable properties [5]. It improves insulin sensitivity, endothelial functions, and inflammation [5]. It has been suggested that thiazoliondione family exert their insulin sensitivity enhancing effect via increasing adiponectin levels [6].

C1q/TNF-Related Protein (CTRP) family is a paralogue of adiponectin and has a favorable effect on insulin sensitivity, inflammation, and lipid metabolism [7]. Several studies have reported the association of these family members with diabetes, coronary artery diseases, non-alcoholic fatty liver disease, poly cystic ovarian syndrome, and metabolic syndrome [8–10]. CTRP3 (also known as cartducin/COR26) is the well-known member of this family with several cardiovascular protective properties [11]. This adipokine activates adenosine monophosphate-activated protein kinase (AMPK) and improves insulin signaling plus insulin sensitivity [12]. Furthermore, CTRP3 reduces secretion of inflammatory cytokines from 3T3-L1 adipocytes [13]. Studies have shown that CTRP3 expression can decline in insulin resistance, where treatment with glucagon-like peptide-1 (GLP-1) receptor agonist enhances its expression and improves insulin sensitivity [14]. In addition, CTRP3 exerts protective effects on lipids metabolism and the cardiovascular system [15].

Several human studies have evaluated circulating levels of CTRP3 in patients with DM, obesity, hypertension, and coronary artery disease [8, 16, 17]. Most of those reported lower levels of CTRP3 in patients with cardio-metabolic diseases. It has been reported that CTRP3 promotes migration and proliferation of endothelial cells [18]. Furthermore, CTRP3 inhibits vascular cell adhesion molecule-1 (VCAM-1) expression and could be a potential biomarker for diabetic retinopathy [19]. Recently, a study by Hu et al, reported a protective role for CTRP3 in cellular model of diabetic nephropathy, they show that CTRP3 attenuates high glucose-induced glomerular mesangial cell dysfunction [20]. Several line of evidence suggested a possible role for CTRP3 in diabetes complications, especially diabetes nephropathy. However, circulating levels of CTRP3 have not been evaluated in patients with diabetic nephropathy so far. Accordingly, the present study aimed to evaluate serum CTRP3 in patients with T2DM and diabetic nephropathy and its association with metabolic parameters.

## Study population and methods

### Study population

This case-control study was conducted on 54 T2DM, 55 T2DM patients with nephropathy (T2DM-NP), and 55 controls. The patients and controls were recruited from Shohahietajrish Hospital, Tehran, Iran, from Jul 2017 to Jun 2018. T2DM was diagnosed based on the criteria of American Diabetes Association [21]. Diabetic nephropathy was diagnosed according to increased urinary albumin excretion (UAE), with patients with UAE > 20 μg/min categorized as T2DM + Nephropathy (T2DM-NP). Regarding to effect of thiazolidinedione and GLP-1 receptor agonist on CTRP3 expression [14, 22], subjects who received these class of medications were excluded from the study. The study was performed according to Declaration of Helsinki and approved by the Ethics Committee of Shahid Beheshti University of Medical Sciences. All study participants signed informed consent forms prior to the study.

### Anthropometric and laboratory measurement

Body mass index (BMI) was calculated by a standard formula (body weight (kg) divided by the square of height (m^2^)). Systolic and diastolic blood pressure were measured by a standard sphygmomanometer after 15 minutes of rest. Five mL of venous blood was captured after an over-night fasting. Serum concentrations of fasting blood glucose (FBG), hemoglobin A1c (HbA1c), low density lipoprotein-cholesterol (LDL-C), total-cholesterol (TC), high density lipoprotein-cholesterol (HDL-C), triglyceride (TG), alanine aminotransferase (ALT), aspartate aminotransferase (AST), and creatinine (Cr) were determined by available commercial kits (Pars Azmoon, Iran). HOMA-IR was calculated by a standard formula. Estimated glomerular filtration rate (eGFR) was calculated by the traditional 4-variable Modification of Diet in Renal Disease (MDRD) equation [23].

### Measuring adipokine and cytokines levels

Serum levels of tumor necrosis factor-α (TNF-α) (Cat #DTA00C) and interleukine-6 (IL-6) (Cat #HS600B) were measured by ELISA kits (R & D Systems, USA) with minimum detectable doses of 1.6 and 0.7 pg/mL, respectively. Adiponectin levels were estimated via ELISA technique (Adipogen, South Korea; Cat #AG-45A-0001YEK-KI01) with inter- and intra-assay coefficient of variations (CV) of 4.4% and 4.6%, respectively. Furthermore, ELISA kit was used to determine serum levels of CTRP3 (Adipogen, South Korea, Cat# AG-45A0042EK-KI01). The intra- and inter-assay CVs for CTRP3 were 7.3% and 5.8%, respectively.

### Statistical analysis

Categorical data were presented with frequency and percentage and further tested by Chi-square test. Continues variables were tested by Kolmogorov-Smirnov for normality. Normally distributed variables were given as mean and standard deviation and tested by student t-test or one-way ANOVA with Bonferroni post hoc analysis. Abnormally distributed data were shown by median and interquartile range (IQR) and tested by Kruskal-Wallis and Bonferroni correction as post hoc analysis. Correlation analysis was conducted by Pearson correlation test. Abnormally distributed data were logarithmically transformed before correlation analysis. Also, multiple linear regression was performed capturing all correlated parameters with CTRP3 in each group. Logistic regression was conducted to assess the association of CTRP3 with diseases status. Finally, receiver operating characteristic (ROC) curve was plotted using binegative exponential model to evaluate the ability of CTRP3 for differentiation between disease statuses. Data analysis was conducted with SPSS 20 (SPSS, USA) and *p* < 0.05 considered as statistical significant.

## Results

### Anthropometric and laboratory data

The details of anthropometric and biochemical measurements are presented in Table 1. Sex, age and BMI showed no significant difference between the studied groups. On the other hand, SBP and DBP were higher in T2DM-NP patients compared to the control group. Furthermore, parameters of glucose and insulin metabolism including FBS, insulin, HOMA-IR and HbA1c showed lower levels in controls compared to T2DM and T2DM-NP groups. TG levels indicated higher levels in T2DM-NP compared to controls, where HDL-C levels were higher in controls compared with other groups. However, TC and LDL-C showed no significant difference between the studied groups. AST demonstrated a higher levels in T2DM and T2DM-NP groups compared to controls, and ALT was higher in T2DM-NP group compared to controls. Furthermore, Cr levels were higher in T2DM-NP compared with control group.

**Table 1:** Anthropometric and biochemical characteristics of study population.

Variables	Control (n = 55)	T2DMs (n = 54)	T2DM-NP (n = 55)	*p* value
Sex [male (%)]	36 (65.5)	32 (59.3)	35 (63.6)	0.790
Age (year)	57.09 ± 7.72	58.96 ± 7.53	57.96 ± 8.56	0.471
BMI (kg/m^2^)	25.65 ± 3.43	27.09 ± 4.06	26.55 ± 4.21	0.155
SBP (mmHg)	127.45 ± 14.96	132.69 ± 19.88	139.84 ± 18.91 ^b^**	0.002
DBP (mmHg)	79 (73–89)	80 (70–92)	85 (80–94)^b^*	0.011
FBG (mg/dL)	93.61 ± 11.45	162.41 ± 22.53 ^a^**	166.95 ± 21.33 ^b^**	<0.001
Insulin (uU/mL)	3.2 (2.1–5.8)	9.4 (5.57–12.35)^a^**	9.6 (7–12.2(^b^**	<0.001
HOMA-IR	0.78 (0.45–1.27)	3.8 (1.99–5.39)^a^**	4 (2.54–5.5 ^b^**	<0.001
HbA1c (%)	4.33 ± 0.93	7.79 ± 1.44 ^a^**	8.01 ± 1.27 ^b^**	<0.001
TG (mg/dL)	122.39 ± 49.42	140.27 ± 42.68	164.04 ± 61.6	<0.001
TC (mg/dL)	172.68 ± 39.78	175.33 ± 45.64	187.2 ± 47.94	0.195
LDL-C (mg/dL)	105.07 ± 32.41	103.74 ± 36.07	115.45 ± 36.28	0.162
HDL-C (mg/dL)	46.13 ± 6.72	41.86 ± 7.46	41.63 ± 5.83 ^b^**	0.001
UAE (μg/min)	11.3 (6.6–13)	10.81 (6.44–14.7)	246.7 (155.56–304.08) ^b^**^,^^c^**	<0.001
eGFR (mL/min/1.73 m^2^)	81.34 ± 25.11	64.35 ± 15.47 ^a^**	30.70 ± 13.78) ^b^**^,^^c^**	<0.001
AST (U/L)	18.61 ± 5.68	21.53 ± 5.69 ^a^*	21.51 ± 6.89 ^b^*	0.018
ALT (U/L)	18.92 ± 7.82	21.43 ± 7.68	23.73 ± 8.22 ^b^**	0.007
Cr (mg/dL)	0.98 ± 0.18	1.18 ± 0.17	2.46 ± 0.78 ^b^**	<0.001
Oral hypoglycemic agent [n (%)]	0	9 (16.7)	12 (21.8)	0.002
Statin use [n (%)]	0	7 (13)	5 (9.1)	0.028

BMI: body mass index, DPB: diastolic blood pressure, SBP: systolic blood pressure, FBG: Fasting blood glucose, HOMA-IR: homeostatic model assessment of insulin resistance, LDL-C: low density lipoprotein-cholesterol, TC: total-cholesterol, HDL-C: high density lipoprotein-cholesterol, TG: triglyceride, UAE: urinary albumin excretion, eGFR: estimated glomerular filtration rate, ALT: alanine aminotransferase, AST: aspartate aminotransferase and Cr: creatinine.

^a.^ Comparison between Control and T2DM

^b.^ Comparison between Control and T2DM-NP

^c.^ Comparison between T2DM and T2DM-NP

* *p* < 0.05

** *p* <0.01

### Serum levels of cytokines and adipokines

**Fig 1:**
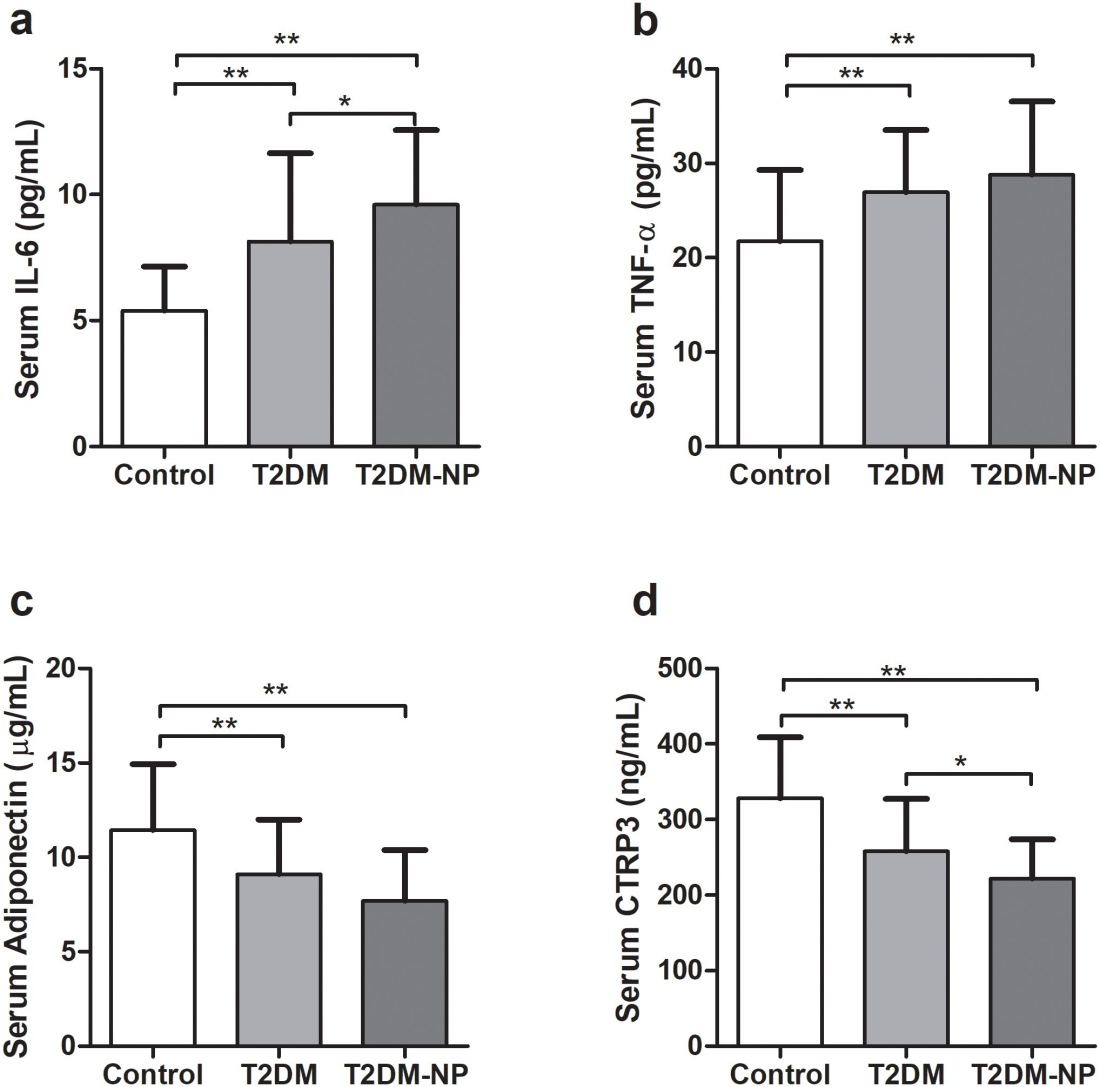
Serum levels of cytokines and adipokines in the studied groups. a) Serum levels of IL-6, b) Serum levels of TNF-α, c) Serum levels of adiponectin and d) Serum levels of CTRP3. * *p* < 0.05 and ** *p* < 0.01.

Serum IL-6 revealed lower concentrations in controls (5.39 ± 1.76 pg/mL) compared to T2DM (8.15 ± 3.5 pg/mL) and T2DM-NP patients (9.60 ± 2.97 pg/mL) (Fig 1a). Meanwhile, T2DM patients demonstrated lower levels of IL-6 compared to T2DM-NP patients. Also, TNF-α serum concentrations were lower in controls (21.76 ± 7.52 pg/mL) compared to T2DM (26.93 ± 6.58 pg/mL) and T2DM-NP (28.78 ± 7.72 pg/mL (Fig 1b). Adiponectin serum levels indicated higher levels in controls (11.45 ± 3.48) compared to T2DM (9.10 ± 2.89) and T2DM-NP (7.70 ± 2.69) groups (Fig 1c). Furthermore, CTRP3 was significantly higher in controls (328.17 ± 80.73 ng/mL) compared to the patients groups, which was higher in T2DM (257.61 ± 69.79 ng/mL) compared to T2DM-NP patients (222.03 ± 51.99 ng/mL) (Fig 1d). In addition, the possible effect of covariates (i. e. age, sex, BMI and medication) was adjusted on serum levels of CTRP3. The results showed that all differences remained significant.

**Fig 2:**
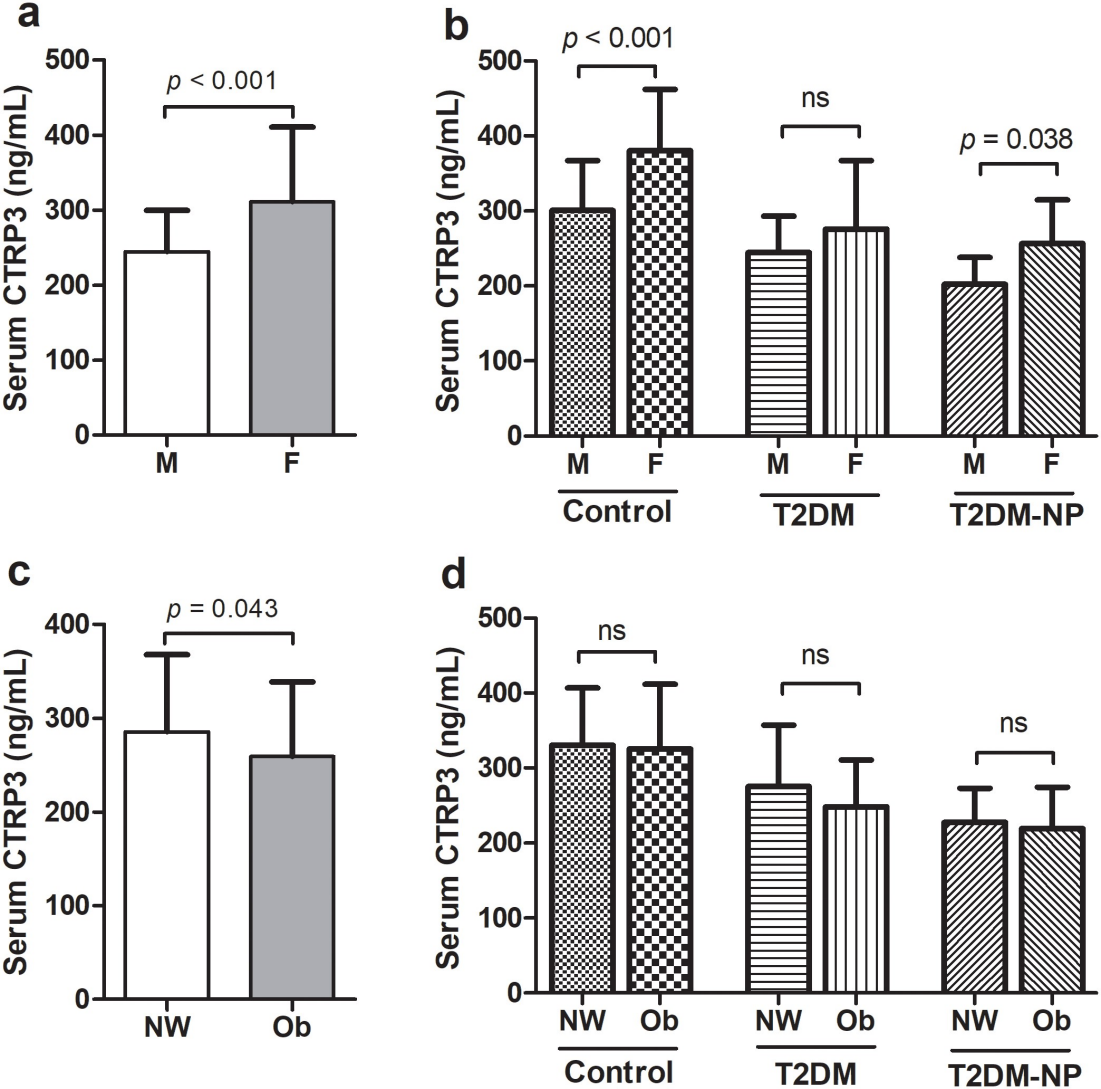
Serum levels of CTRP3 stratified by sex and BMI. a) Serum levels of CTRP3 stratified by sex, b) Serum levels of CTRP3 stratified by sex in the each group, c) Serum levels of CTRP3 stratified by BMI and d) Serum levels of CTRP3 stratified by BMI in each group. M: Male, F: Female, NW: Normal weight and Ob: Obese.

In addition, CTRP3 were higher in female (311.38 ± 99.37 ng/mL) compared to male (244.45 ± 55.36 ng/mL, *p* < 0.001) (Fig 2a). Furthermore, CTRP3 serum levels were higher in female compared with male in controls (380.12 ± 81.80 vs. 300.85 ± 65.15 ng/mL, *p* < 0.001) and T2DM-NP (256.57 ± 58.30 vs. 202.34 ± 35.93 ng/mL, *p* = 0.038) groups, however in T2DM group there was no significant difference (276.03 ± 90.95 vs. 245.09 ± 48.17 ng/mL, *p* = 0.65) (Fig 2b).

We analyzed CTRP3 levels stratified by BMI (normal weight: BMI ≤25 and overweight/obese: BMI >25). The results indicated that CTRP3 were higher in normal weight (285.60 ± 81.99 ng/mL) compared to overweigh/obese (259.21 ± 79.56 ng/mL, *p* = 0.043) (Fig 2c). However, there was no significant difference between obese and normal weight in the studied groups (Fig 2d).

Association of serum CTRP3 with risk of diseases status was examined by multinomial logistic regression and the results showed independent association of CTRP3 levels with risk of T2DM and T2DM-NP (Table 2).

**Table 2:** Odd ratio for T2DM and T2DM-NP per 10 ng/mL increase in serum levels of CTRP3.

	Odd ration (95% confidence interval)	*p* value
Crude models		
T2DM	0.882 (0.831–0.937)	<0.001
T2DM-NP	0.792 (0.732–0.857)	<0.001
Adjusted models*		
T2DM	0.804 (0.731–0.883)	<0.001
T2DM-NP	0.730 (0.654–0.814)	<0.001

Reference group: Control

*Adjusted for age, sex, BMI and adiponectin.

**Fig 3:**
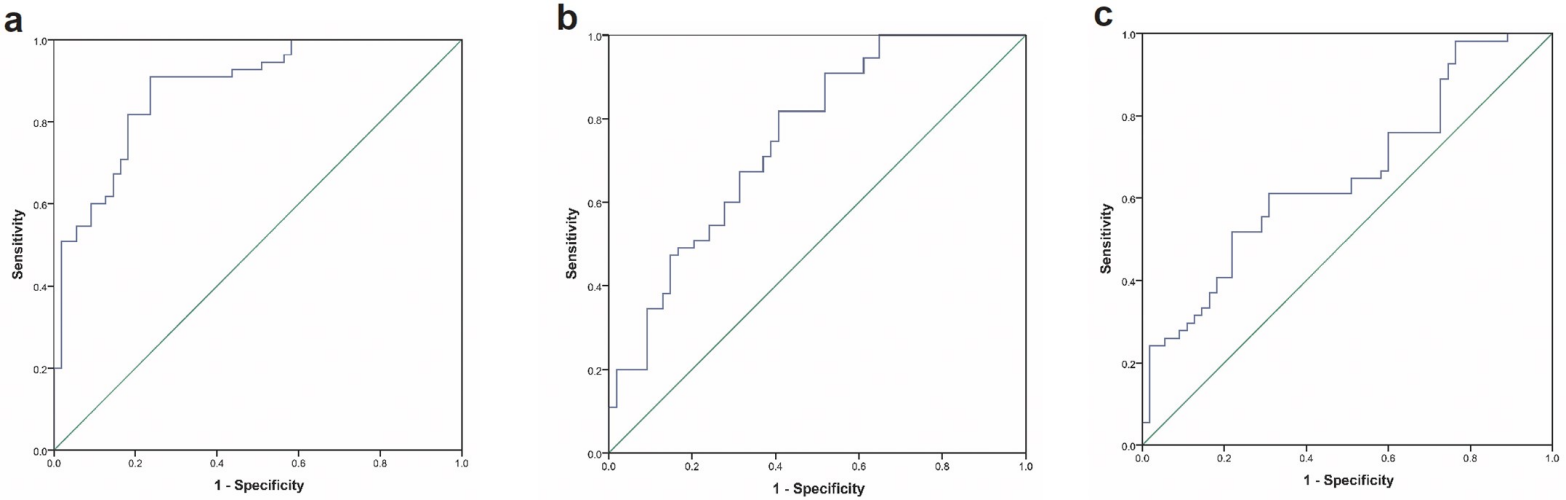
ROC curve for discrimination between a) T2DM-NP and control, b) T2DM and control and c) T2DM and T2DM-NP.

Further, ROC analysis demonstrated that CTRP3 had a good ability for differentiation between control and T2DM-NP patients (area under curve (95% confidence interval): 0.881 (0.820–0.943) and p < 0.001) (Fig 3a). Nevertheless, ability for differentiation between controls and T2DM was not as high as that for T2DM patients (area under curve (95% confidence interval): 0.751 (0.661–0.841) and p < 0.001) (Fig 3b). Furthermore, CTRP3 was not a good factor for differentiation between T2DM and T2DM-NP groups (area under curve (95% confidence interval): 0.656 (0.553–0.758) and p = 0.005) (Fig 3c).

### Association of CTRP3 with anthropometric and biochemical parameters

Correlation analyses were performed in all studied groups with the results presented in Table 3. In the control group, CTRP3 was negatively correlated with insulin and HOMA-IR. Furthermore, in T2DM and T2DM-NP groups, CTRP3 demonstrated a negative correlation with insulin, HOMA-IR, eGFR, IL-6 and TNF-α, but a positive correlation with adiponectin. In addition, CTRP3 negatively correlated with Cr in T2DM-NP patients. Multiple linear regression revealed that CTRP3 independently associated with HOMA-IR (B [standard error] = -64.33 [23.02] and p = 0.007) and adiponectin (B [standard error] = 9.21 [2.63] and *p* = 0.001) in T2DM group as well as eGFR (B [standard error] = 0.953 [0.441] and *p* = 0.036) and HOMA-IR (B [standard error] = -62.773 [25.17] and *p* = 0.016) in T2DM-NP patients.

**Table 3:** Correlation of CTRP3 with anthropometric and biochemical variables in the studied groups.

Variables	Control	T2DM	T2DM-NP
Age	-0.049	0.143	0.135
BMI	-0.254	-0.154	-0.240
SBP	-0.084	0.022	-0.075
Log DBP	-0.044	-0.122	-0.117
FBG	-0.250	-0.229	-0.205
Log Insulin	-0.415**	-0.312*	-0.407**
Log HOMA	-0.438**	-0.327*	-0.436**
HbA1c	-0.178	-0.286*	-0.287*
TG	-0.068	-0.056	-0.057
TC	0.008	-0.040	0.109
LDL-C	0.089	-0.085	0.119
HDL-C	0.012	0.206	0.100
Cr	-0.031	-0.175	-0.389**
eGFR	0.149	0.274*	0.428**
Log UAE	0.083	0.185	-0.255
AST	-0.173	-0.178	-0.079
ALT	-0.057	0.005	0.015
IL-6	-0.089	-0.342*	-0.386**
TNF-α	0.089	-0.359**	-0.365**
Adiponectin	-0.038	0.436**	0.273*

BMI: body mass index, DPB: diastolic blood pressure, SBP: systolic blood pressure, FBG: Fasting blood glucose, HOMA-IR: homeostatic model assessment of insulin resistance, HbA1c: hemoglobin A1c, LDL-C: low density lipoprotein-cholesterol, TG: triglyceride, TC: total-cholesterol, HDL-C: high density lipoprotein-cholesterol, Cr: creatinine, eGFR: estimated glomerular filtration rate, UAE: urinary albumin excretion, ALT: alanine aminotransferase, AST: aspartate aminotransferase, IL-6: interleukine-6 and TNF-α: tumor necrosis factor-α.

* *p* < 0.05

** *p* <0.01

## Discussion

CTRP3 is the most studied member of CTRP family and several studies evaluated its circulating levels in the context of cardio-metabolic diseases [10, 16, 17, 19]. Nevertheless, circulating levels of this adipokine have not been studied in patients with kidney diseases. Most previous studies reported lower levels of CTRP3 in patients with T2DM [8, 24, 25]; however, a study by Choi et. al. showed higher levels of CTRP3 in patients with T2DM [26]. They developed an ELISA for measuring CTRP3 that is different from the ELISA kit was used in the present study. The difference between methods used for measuring CTRP3 could be a possible cause for this contradiction. Furthermore, several studies also indicated in vivo association between CTRP3 and parameters of glucose metabolism [27]. Ban et. al. reported lower levels of CTRP3 in newly diagnosed T2DM patients [24], and Deng et al. showed lower concentrations of CTRP3 in obesity and hypertension [27]. In line with previous studies, in the present study, CTRP3 serum levels were significantly lower in patients with T2DM. Furthermore, CTRP3 was negatively correlated with insulin and HOMA-IR across all studied groups. Various studies suggested a negative correlation between HOMA-IR and CTRP3 in patients with cardio-metabolic diseases [3, 8, 25]. Lower levels of CTRP3 in T2DM and negative correlation between CTRP3 and HOMA-IR could be caused by the effect of insulin resistance on the expression of this adipokine in adipose tissue. Confirming this statement, previous studies revealed that insulin resistance reduced the expression of CTRP3. On the other hand, CTRP3 exerts multiple effects on insulin and glucose metabolism [11]. CTRP3 lowers glucose levels in mice and suppresses gluconeogenesis genes [12]. In addition, it has been revealed that CTRP3 improves insulin sensitivity by reducing inflammation in 3T3-L1 adipocytes [13]. Furthermore, CTRP3 enhances protein kinase B (PKB) and AMPK phosphorylation and promote expression of phosphoinositide 3-kinase (PI3K) and Glucose transporter type 4 (GLUT-4), improving insulin sensitivity in adipocytes [11]. Therefore, the reduced CTRP3 seems to be due to insulin resistance and might be a factor which exacerbates insulin resistance in patients with T2DM.

In the present study CTRP3 showed a higher levels in female compared to male which is in agreement with previous studies which reported sex specific pattern for CTRP3 expression and serum levels in animal and human studies [7, 8, 17]. These results suggested that CTRP3 might be controlled by hormonal status, however more studies are needed in this regard.

For the first time, our results demonstrated lower levels of CTRP3 in patients with diabetic nephropathy compared with healthy controls and those with T2DM. In the present study, CTRP3 revealed a good sensitivity and specificity for differentiation between controls and diabetic nephropathy. Also, CTRP3 showed a significant correlation with markers of kidney function. CTRP3 was negatively associated Cr and positively associated with eGFR. Previous studies indicated an association between other members of CTRP family and endothelial functions. For instance, CTRP9 demonstrated a link with markers of endothelial function in patients with T2DM and coronary artery disease [28]. A study by Yan et al. showed lower levels of CTRP3 in patients with diabetic retinopathy [19]. They found that CTRP3 downregulates VCAM-1 expression via AMPK pathway [19]. It has been reported that CTRP3 promotes migration and proliferation of endothelial cells [18]. In addition, Hu et. al. reported a protective role for CTRP3 in high glucose-induced glomerular mesangial cell dysfunction that was suggested as a cellular model of diabetic nephropathy [20]. CTRP3 exert its protective role via inactivation of janus kinase 2/signal transducers and activators of transcription 3 (JAK2/STAT3) in high glucose-induced glomerular mesangial cell [20]. The association between CTRP3 and Cr as well as eGFR are in vivo evidence for association of CTRP3 with diabetic nephropathy that confirm previous study by Hu et. al [20].

Meanwhile, inflammation could be an effective factor in endothelial dysfunction and complication of diabetes. CTRP3 reduces production of TNF-α and IL-6 in adipocytes [13]. In this regard, a negative correlation between CTRP3 and inflammatory markers has been reported in patients with T2DM and coronary artery disease [8]. In the present study, a negative correlation was observed between inflammatory markers and CTRP3.

With regards to the favorable effect of CTRP3 on the mechanism of T2DM development and its complications such as insulin resistance, inflammation, and endothelial dysfunction, reduced levels of CTRP3 could be a factor that exacerbates pathogenic conditions in patients with T2DM. This intensifies diabetes complication especially microvascular complication. However, the present study had a cross-sectional design and limited us to conclude a causal relationship between decreased serum levels of CTPR3 and diabetic nephropathy. In this regard, future studies are needed to prove this concept.

## Supporting information

S1 File Datasets supporting the conclusions of this article. (XLSX)
